# Penfluridol suppresses pancreatic tumor growth by autophagy-mediated apoptosis

**DOI:** 10.1038/srep26165

**Published:** 2016-05-18

**Authors:** Alok Ranjan, Sanjay K. Srivastava

**Affiliations:** 1 Department of Biomedical Sciences and Cancer Biology Center, Texas Tech University Health Sciences Center, Amarillo, TX 79106, USA

## Abstract

Pancreatic tumors exhibit enhanced autophagy as compared to any other cancer, making it resistant to chemotherapy. We evaluated the effect of penfluridol against pancreatic cancer. Penfluridol treatment induced apoptosis and inhibited the growth of Panc-1, BxPC-3 and AsPC-1, pancreatic cancer cells with IC_50_ ranging between 6–7 μM after 24 h of treatment. Significant autophagy was induced by penfluridol treatment in pancreatic cancer cells. Punctate LC3B and autophagosomes staining confirmed autophagy. Inhibiting autophagy by chloroquine, bafilomycin, 3-methyladenine or LC3BsiRNA, significantly blocked penfluridol-induced apoptosis, suggesting that autophagy lead to apoptosis in our model. Penfluridol treatment suppressed the growth of BxPC-3 tumor xenografts by 48% as compared to 17% when treated in combination with chloroquine. Similarly, penfluridol suppressed the growth of AsPC-1 tumors by 40% versus 16% when given in combination with chloroquine. TUNEL staining and caspase-3 cleavage revealed less apoptosis in the tumors from mice treated with penfluridol and chloroquine as compared to penfluridol alone. Penfluridol treatment also suppressed the growth of orthotopically implanted Panc-1 tumors by 80% by inducing autophagy-mediated apoptosis in the tumors. These studies established that penfluridol inhibits pancreatic tumor growth by autophagy-mediated apoptosis. Since penfluridol is already in clinic, positive findings from our study will accelerate its clinical development.

Pancreatic cancer is the fourth leading cause of cancer-related deaths in the United States. It is mostly detected at late stage and get resistant to chemotherapies^1^. Pancreatic tumors have higher basal state autophagy as compared to other epithelial tumors^1^. Autophagy is a highly conserved process by which, cells get rid of unwanted cellular components like unfolded proteins through lysosomal degradation^1^. It is a dynamic process in which, useless cellular components gets sequestered in double membranous vesicles called autophagosomes. Autophagosomes fuses with lysosomes forming autolysosomes, which results in digestion of enclosed materials by acidic enzymes^2^. Elevated autophagy in pancreatic tumors is also responsible for drug resistance^3^. It has been reported that anti-cancer agents like gemcitabine inhibits the growth of pancreatic cancer by inducing autophagy^4^. Pancreatic cancer shows initial sensitivity to gemcitabine therapy but rapidly develops resistance^5^.

Recently, few studies indicated that schizophrenia patients taking anti-psychotic medications were less prone to cancer^6^. It has been suggested that antipsychotic agents like chlorpromazine and thioridazine induces autophagy-mediated cell death in glioma cells^7^. However, these drugs have been reported to have fatal toxicities as well^8^,^9^. Penfluridol is an anti-psychotic drug, clinically available since 1970 for the treatment of schizophrenia^10^. Limited studies have shown anti-cancer properties of penfluridol; however, further studies are required to evaluate its anti-cancer effects against pancreatic cancer^11^.

In the present study, we investigated the growth suppressive effects of penfluridol in pancreatic cancer. Significant suppression of pancreatic cancer cells growth was observed by penfluridol treatment. Penfluridol also induced significant autophagy and apoptosis, which was blocked by autophagy inhibitors. Oral administration of penfluridol significantly suppressed the growth of pancreatic tumors in xenograft and orthotopic models by inducing autophagy-mediated apoptosis. To the best of our knowledge, this is the first report of autophagy-mediated apoptosis leading to pancreatic tumor growth suppression by penfluridol.

## Results

### Penfluridol suppresses the survival of pancreatic cancer cells

**Figure 1:**
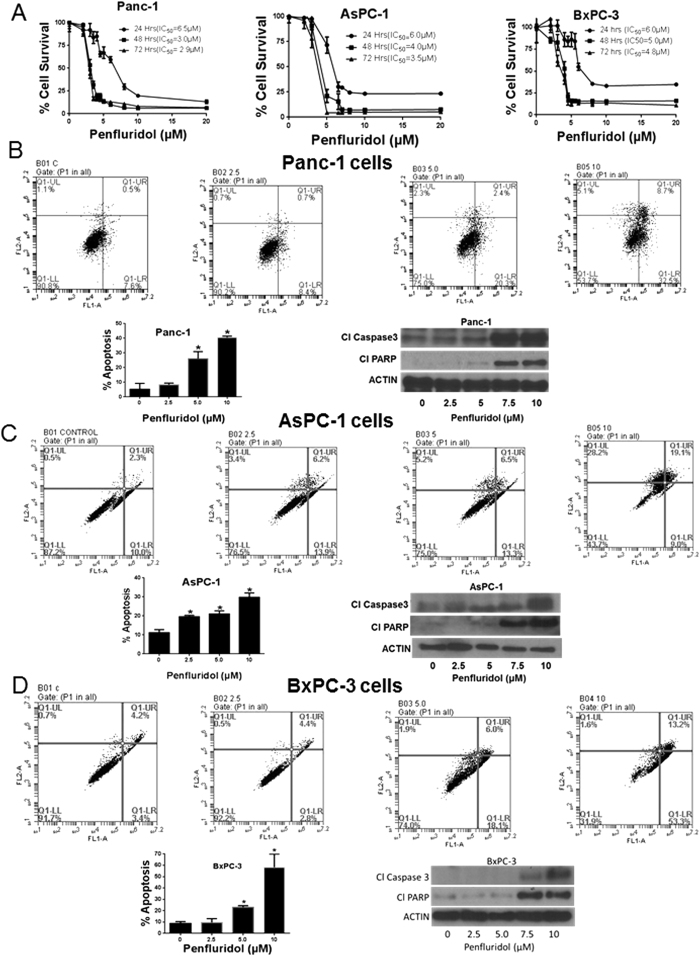
Penfluridol induces apoptosis and suppresses survival of pancreatic cancer cells. (**A**) Panc-1, AsPC-1 and BxPC-3 cells were treated with different concentrations of penfluridol for 24, 48 and 72 h. Cell survival was measured by Sulforhodamine B assay to estimate IC_50_ values. The experiments were repeated three times with 8 replicates in each experiment. (**B–D**) Approximately 0.3 × 10^6^ Panc-1, AsPC-1 and BxPC-3 cells were plated in 6 well plates, treated with 2.5, 5.0 and 10 μM penfluridol for 24 h and processed for AnnexinV/FITC apoptosis assay using Accuri C6 flow cytometer. Values were plotted as means ± SD. Experiment was repeated three times. *Statistically significant at p ≤ 0.05 when compared with control. Panc-1, AsPC-1 and BxPC-3 cells were treated with varying concentrations of penfluridol for 24 h and processed for western blotting. Representative blots showing concentration-dependent effect of penfluridol treatment on Cl Caspase 3 and Cl PARP. Actin was used as loading control. Shown figure is the representative blots of at least three independent experiments.

The cytotoxicity of penfluridol was determined in pancreatic cancer cells by Sulforhodamine B (SRB) assay. Treatment of Panc-1, AsPC-1 and BxPC-3 cells with varying concentrations of penfluridol resulted in significantly reduced survival of cells in a concentration and time-dependent manner (Fig. 1A). The IC_50_ of penfluridol was 6.5 μM, 3 μM and 2.9 μM in Panc-1 cells following 24, 48 and 72 h of treatment respectively (Fig. 1A). Similarly, in AsPC-1 cells, IC_50_ was 6 μM, 4 μM and 3.5 μM (Fig. 1A) and in BxPC-3 cells was 6 μM, 5 μM and 4.8 μM (Fig. 1A) after 24, 48 and 72 h of treatment respectively. These results suggest potential cytotoxic effects of penfluridol in pancreatic cancer cells.

### Induction of apoptosis by penfluridol

To determine the mechanism of growth suppressive effects of penfluridol, Annexin/FITC apoptosis assay was performed by flow cytometer in Panc-1, AsPC-1 and BxPC-3 pancreatic cancer cells. As shown in Fig. 1B, 24 h treatment with penfluridol resulted in significant apoptosis in Panc-1 cells. The percent apoptosis in Panc-1 cells increased to about 26% with 5 μM penfluridol and to 40% with 10 μM penfluridol, after 24 h of treatment (Fig. 1B). Cleavage of caspase 3 and PARP was also observed in Panc-1 cells confirming induction of apoptosis by penfluridol (Fig. 1B). Similarly, around 20% and 30% apoptosis was observed in AsPC-1 cells after 24 h treatment with 5 μM and 10 μM penfluridol respectively (Fig.1C). Induction of apoptosis by penfluridol treatment in AsPC-1 cells was confirmed by cleavage of caspase 3 and PARP (Fig. 1C). About 23% and 58% apoptosis was observed in BxPC-3 cells after 24 h of treatment with 5 and 10 μM of penfluridol respectively (Fig. 1D).

### Penfluridol induces autophagy in pancreatic cancer cells

**Figure 2:**
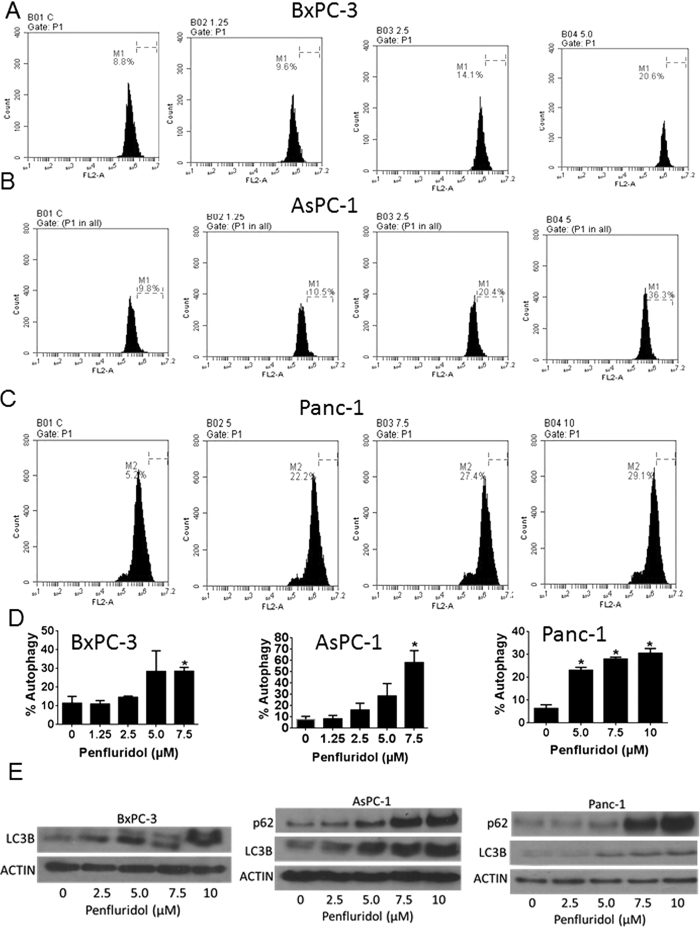
Induction of autophagy with penfluridol treatment. (**A–D**) BxPC-3, AsPC-1 and Panc-1 cells were plated in six well plates and treated with different concentration of penfluridol for 24 h. Cells were stained with 0.4 μg/ml acridine orange and evaluated by Accuri C6 flow cytometer. Values were plotted as means ± SD. Experiment was repeated three times. *Statistically significant when compared with control at p ≤ 0.05. (**E**) BxPC-3, AsPC-1 and Panc-1 cells were treated with different concentration of penfluridol for 24 h. Representative blots showing concentration-dependent effect of penfluridol on p62 and LC3B expression. Actin was used as loading control. Figure shown is the representative blots of at least three independent experiments.

**Figure 3:**
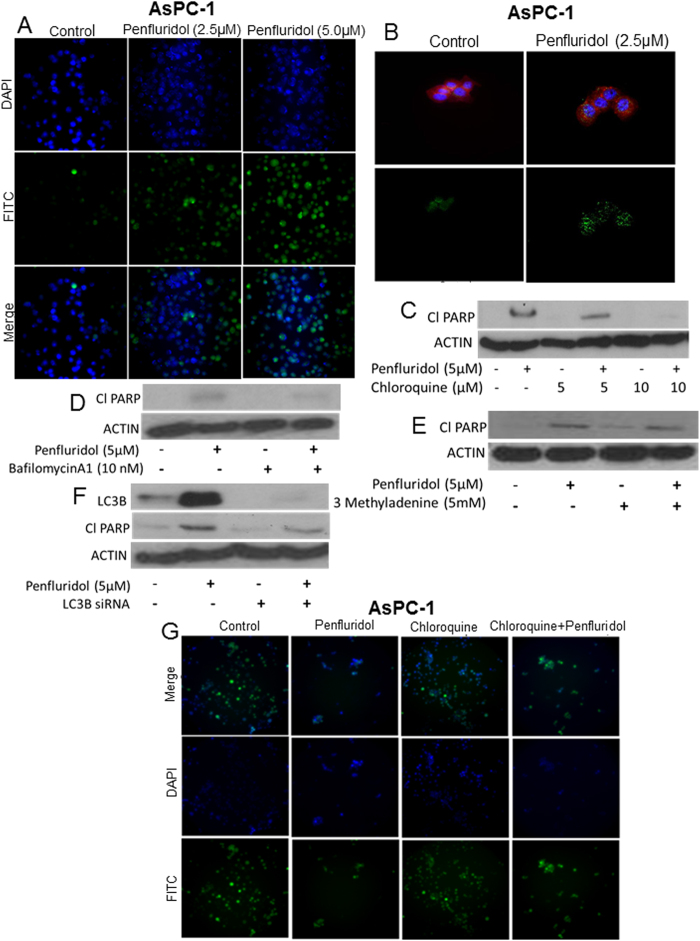
Penfluridol induces autophagy-mediated apoptosis. (**A**) AsPC-1 cells were treated with 2.5 and 5.0 μM penfluridol. After 24 h of penfluridol treatment, cells were stained with NucBlue followed by staining with 0.4 μg/ml acridine orange. Images were taken immediately using florescence microscopy. Green florescence represents acridine orange whereas blue represent DAPI. (**B**) AsPC-1 cells were plated on coverslip. Cells were treated with 2.5 μM penfluridol for 24 h. Cells were processed, mounted on slides and images were taken using florescence microscopy. Green florescence represents LC3B, blue represents DAPI, whereas red represents actin. AsPC-1 cells were treated with penfluridol (5 μM) for 24 h after (**C**) cells were pretreated with 5 and 10 μM chloroquine for 3 h (**D**) cells were pretreated with 10 nM BafilomycinA1 for 3 h (**E**) Cells were pretreated with 5 mM 3-methyladenine for 3 h (**F**) AsPC-1 cells were transfected with LC3B siRNA. Levels of LC3B and Cl PARP were evaluated by western blotting. Actin was used as loading control. (**G**) AsPC-1 cells were plated in six well plates and treated with 10 μM chloroquine for 3 h followed by treatment with 5 μM penfluridol for 24 h. Cells nucleus were stained with NucBlue for 20 minutes. Images were taken immediately after adding 50 nM lysotracker green DND-26. Green florescence represents lysosomes whereas blue represent DAPI for nucleus.

Since we observed significant apoptosis by penfluridol treatment in pancreatic cancer cells, we next wanted to investigate the mechanism by which penfluridol treatment induced apoptosis. Acridine orange assay suggested that penfluridol induced autophagy in a concentration-dependent manner in BxPC-3, AsPC-1 and Panc-1 cells (Fig. 2A–C). BxPC-3 cells when exposed to 7.5 μM penfluridol induced about 30% autophagy (Fig. 2D). Around 58% and 28% autophagy was observed in AsPC-1 and Panc-1 cells respectively, when treated with 7.5 μM penfluridol (Fig. 2D). Western blot results showed substantial up-regulation of LC3B, a marker for the formation of autophagic vesicles as well as enhanced expression of p62 in AsPC-1 and Panc-1 cells (Fig. 2E). Autophagy induction by penfluridol was further confirmed by microscopy after staining pancreatic cancer cell with acridine orange. Significant increase in green staining for acridine orange was observed in AsPC-1 cells after 24 h of treatment with 2.5 μM and 5.0 μM penfluridol (Fig. 3A). Immunoflorescence showed punctate LC3B as represented by green staining in AsPC-1 cells after treatment with 2.5 μM penfluridol for 24 h, confirming autophagy induction by penfluridol in pancreatic cancer cells (Fig. 3B).

### Autophagy inhibitors blocks penfluridol-induced apoptosis

To further delineate the mechanism of apoptosis by penfluridol treatment in pancreatic cancer cells, we inhibited autophagy by pretreatment of cells with known autophagy inhibitors like chloroquine, bafilomycinA1 or 3-methyladenine followed by treatment with 5 μM penfluridol for 24 h. Blocking autophagy resulted in reduced apoptosis as indicated by less cleavage of PARP with penfluridol treatment in AsPC-1 cells (Fig. 3C–E). These results indicate that penfluridol treatment suppresses the growth of pancreatic cancer cells by inducing autophagy-mediated apoptosis.

### Silencing LC3B reduces the effect of penfluridol

To confirm the role of autophagy in penfluridol-induced apoptosis in pancreatic cancer cells, we knocked down LC3B using LC3BsiRNA before treatment with penfluridol. LC3BsiRNA silenced 94% of LC3B in AsPC-1 cells (Fig. 3F). Our results demonstrated that silencing LC3B reduced cleavage of PARP by penfluridol treatment in AsPC-1 cells (Fig. 3F). Taken together these results confirmed that penfluridol treatment induced autophagy-mediated apoptosis in pancreatic cancer cells.

### Penfluridol treatment results in autolysosomes formation

To further evaluate induction of autophagy by penfluridol treatment, we tracked the lysosomes in AsPC-1 cells, using a commercial kit. Autophagy in cells tends to decrease the number of lysosomes, as during autophagy, autophagosomes fuses with lysosomes forming autolysosomes. Treatment of AsPC-1 cells with 5 μM penfluridol for 24 h reduced the number of lysosomes, as observed by reduced staining for green DND-26, which stains lysosomes (Fig. 3G). However, we observed less autophagy in the cells treated with 5 μM penfluridol for 24 h after pretreatment with 10 μM chloroquine (lysosomotrophic agents), as compared to penfluridol treatment alone (Fig. 3G). We also noticed that penfluridol treatment in combination with chloroquine increased the survival of cells, as compared to penfluridol treatment alone (Fig. 3G).

### Penfluridol suppresses the growth of subcutaneously implanted pancreatic tumors by autophagy-mediated apoptosis

**Figure 4:**
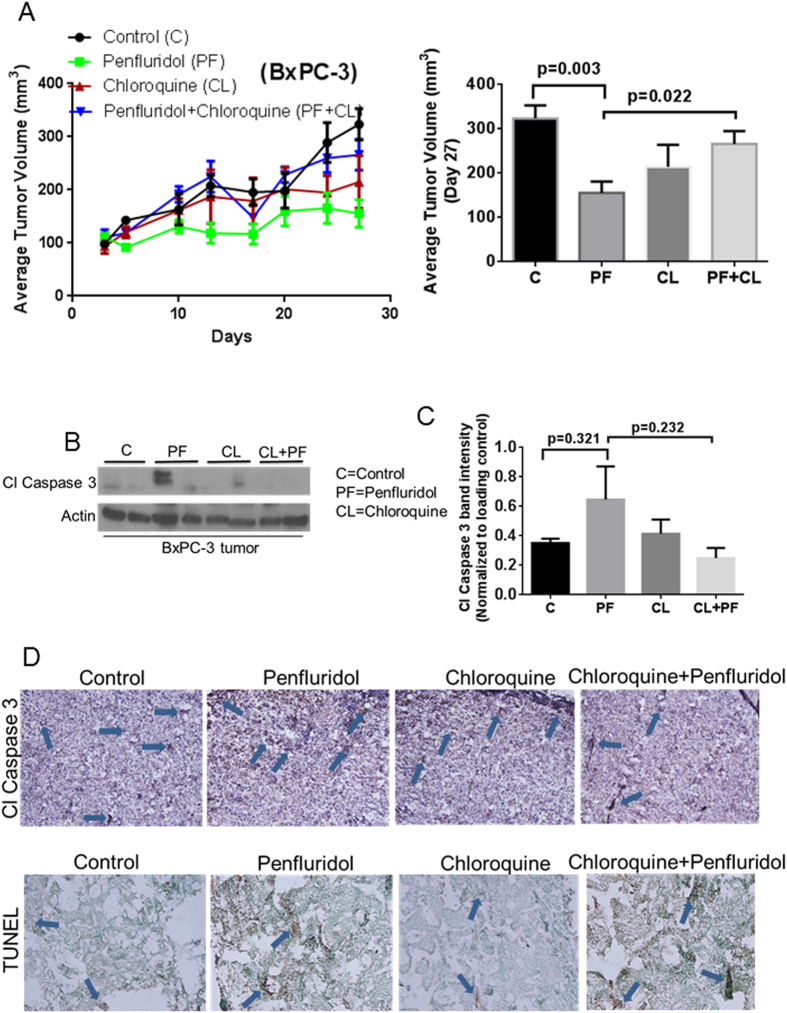
Penfluridol suppresses the growth of subcutaneously implanted BxPC-3 pancreatic tumors by autophagy-mediated apoptosis. (**A**) About 1 × 10^6^ BxPC-3 pancreatic cancer cells were injected subcutaneously in flanks of 4–6 week old athymic nude mice. Once tumor volume reached around 70 mm^3^, mice were randomly divided into 4 groups. Group I received vehicle only and served as control. Group II received 10 mg/kg penfluridol by oral gavage every day. Group III received 50 mg/kg chloroquine (i.p) every day whereas Group IV received 10 mg/kg penfluridol as well as 50 mg/kg chloroquine every day till day 27. Tumors volume was measured twice a week using vernier caliper. Values were plotted as mean ± SEM. Statistically different at p ≤ 0.05 (**B**) Subcutaneously implanted tumors were removed aseptically after terminating the experiments. Tumors were homogenized, lysed and analyzed for Cl Caspase 3. Actin was used as loading control. Each lane of blot represents tumor from individual mice. (**C**) Blots were quantitated, normalized with actin and represented as bars. Values were plotted as means ± SEM and considered statistically significant at p ≤ 0.05 (**D**) Tumors were sectioned and immunostained for Cl Caspase 3 and TUNEL as described in method section.

**Figure 5:**
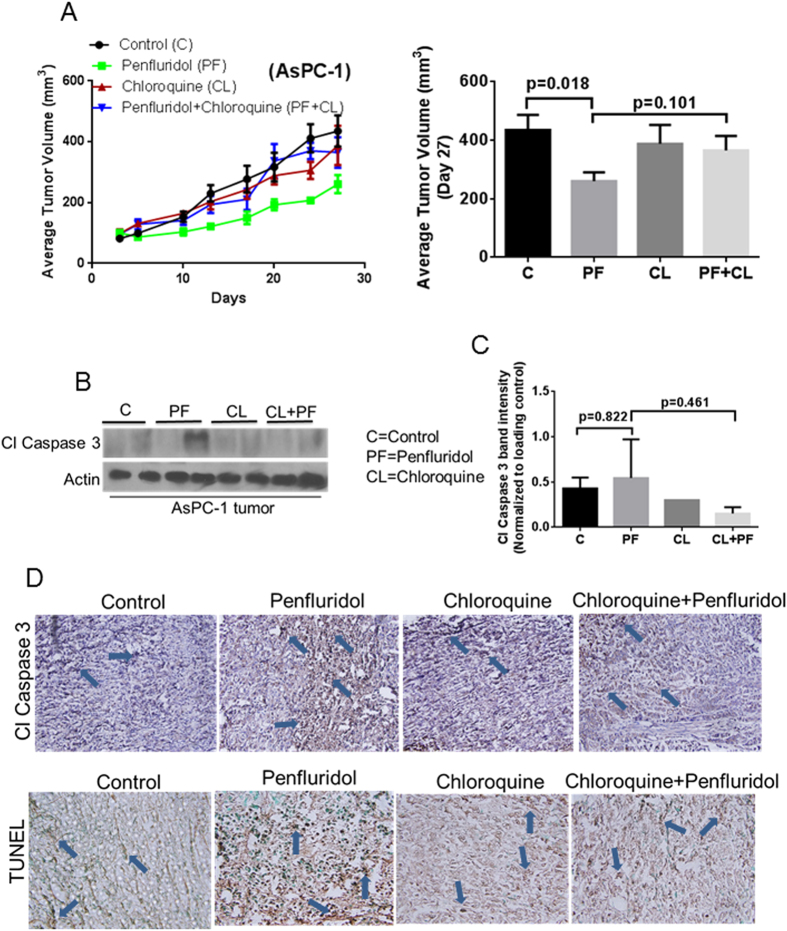
Penfluridol suppresses the growth of subcutaneously implanted AsPC-1 pancreatic tumors by autophagy-mediated apoptosis. (**A**) About 1 × 10^6^ AsPC-1 pancreatic cancer cells were injected subcutaneously in flank of 4–6 week old athymic nude mice. Once tumor volume reached around 70 mm^3^, mice were randomly divided into 4 groups. Group I received vehicle only and served as control. Group II received 10 mg/kg penfluridol by oral gavage every day. Group III received 50 mg/kg chloroquine (i.p) every day whereas Group IV received 10 mg/kg penfluridol as well as 50 mg/kg chloroquine every day till day 27. Tumors volume was measured twice a week using vernier caliper. Values were plotted as mean ± SEM. Statistically different at p ≤ 0.05. (**B**) Subcutaneously implanted tumors were removed aseptically after terminating the experiments. Tumors were homogenized, lysed and analyzed for Cl Caspase 3. Actin was used as loading control. Each lane of blot represents tumor from individual mice. (**C**) Blots were quantitated, normalized with actin and represented as bars. Values were plotted as means ± SEM and considered statistically significant at p ≤ 0.05. (**D**) Tumors were sectioned and immunostained for Cl Caspase 3 and TUNEL as described in method section.

To evaluate the efficacy of penfluridol treatment *in vivo* and to establish the mechanism of tumor growth inhibition in pancreatic cancer, BxPC-3 and AsPC-1 cells were implanted subcutaneously in athymic nude mice. After the tumors reached about 70 mm^3^ in size, mice were treated with 10 mg/kg penfluridol by oral gavage every day and the experiment was terminated at day 27. Mice were sacrificed at day 27 due to tumor burden. Our results showed that penfluridol treatment suppressed the growth of BxPC-3 tumors by 48% and AsPC-1 tumors by 40% (Figs 4A and 5A). In another experiment, 50 mg/kg chloroquine was given to mice intraperitoneally every day alone or in combination with 10 mg/kg penfluridol by oral gavage every day. Chloroquine is an inhibitor of autophagy. Penfluridol treatment in combination with chloroquine resulted in only 17% and 16% growth suppression of BxPC-3 and AsPC-1 tumors respectively (Figs 4A and 5A). These results clearly suggest that penfluridol treatment suppresses the growth of pancreatic tumors by inducing autophagy.

Tumors collected after humanely sacrificing the mice were subjected to western blot analysis. Induction of apoptosis in tumors was also confirmed by TUNEL and cleaved caspase 3 staining by immunohistochemistry. In agreement with our *in vitro* observations, our *in vivo* results demonstrated increased staining for cleaved caspase 3 with penfluridol treatment alone as compared to when penfluridol was combined with chloroquine (Figs 4B–D and 5B–D). Similarly, we observed less apoptosis in the tumors from mice treated with combination of penfluridol and chloroquine as compared to penfluridol treatment alone, as evaluated by TUNEL staining (Figs 4D and 5D). These results indicated that pancreatic tumor growth suppression by penfluridol was associated with induction of autophagy leading to apoptosis.

### Penfluridol suppresses the growth of orthotopic pancreatic tumor

**Figure 6:**
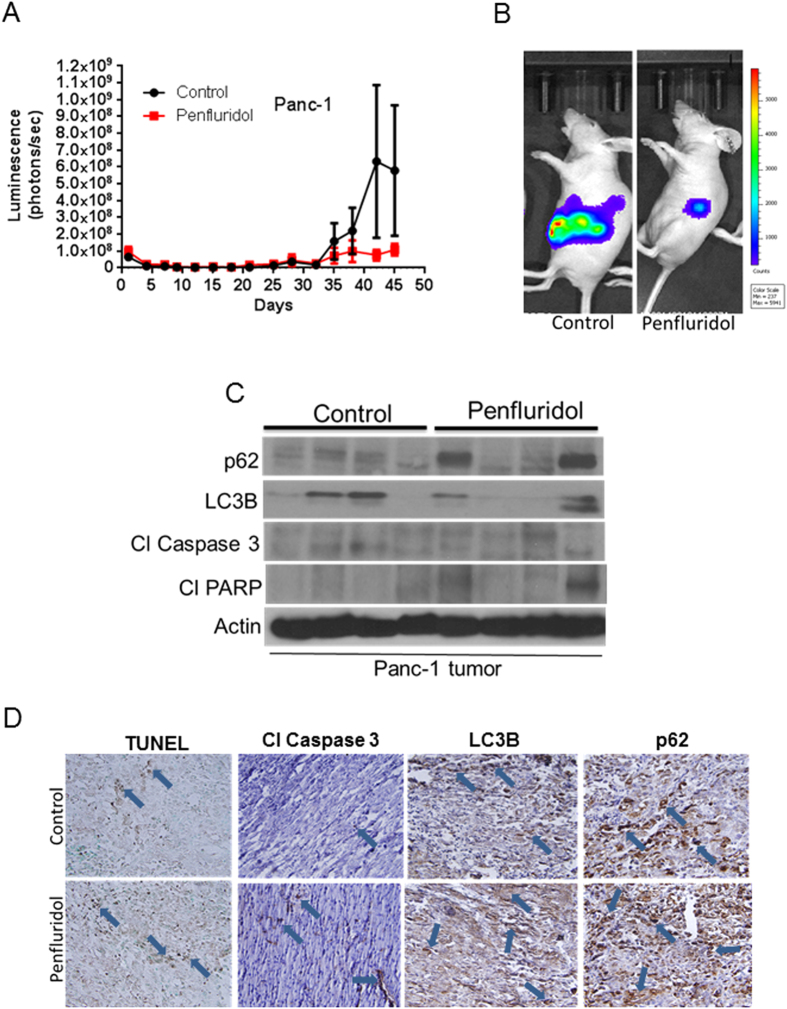
Penfluridol suppresses the growth of orthotopically implanted pancreatic tumor. About 1 × 10^6^ Panc-1-luc cells were implanted surgically on the pancreas of 4–6 week old athymic nude mice. Mice were treated with 10 mg/kg penfluridol starting day 9^th^ after tumor cells implantation till day 45. (**A**) Tumor luminescence (photons/second) was measured about thrice a week and plotted against days. (**B**) Representative mouse from control and penfluridol treated group. After terminating the experiment; pancreas with tumors were removed aseptically, lysed and analyzed for p62, LC3B, Cl Caspase 3 and Cl PARP by western blotting. Actin was used as loading control (**C**) Each lane of blot represents tumor from separate mouse. (**D**) Pancreas with tumors were sectioned and immunostained for p62, LC3B, Cl Caspase 3 as well as stained for TUNEL.

To further validate and as a proof of concept, we implanted Panc-1 luc cells orthotopically into the pancreas of athymic nude mice. After eight days of tumor cell implantation, mice were treated with 10 mg/kg penfluridol every day by oral gavage. Growth of orthotopically implanted pancreatic tumors was suppressed by 80% by penfluridol treatment (Fig. 6A). The experiment was terminated and pancreas with tumors was removed aseptically. A part of pancreatic tissue with tumors was snap frozen for western blotting whereas other part was fixed for immunohistochemical staining and TUNEL assay. In accordance with our *in vitro* finding, our results showed that penfluridol treatment induced autophagy and apoptosis as observed by increase in p62 and LC3B expression and increased cleavage of caspase3 and PARP in the tumors from penfluridol treated mice (Fig. 6C). The tumors were also examined by immunohistochemistry and TUNEL assay. Consistent with our *in vitro* results, tumors from penfluridol treated group exhibited more cleavage of caspase3 as well as enhanced expression of LC3B and p62, as compared to control (Fig. 6D). Furthermore, we observed increased TUNEL staining with penfluridol treatment confirming induction of apoptosis in tumor tissue (Fig. 6D). These results clearly demonstrated efficacy of penfluridol in inhibiting the growth of pancreatic tumors by inducing autophagy and apoptosis.

### Penfluridol treatment does not cause any major side effects in a chronic toxicity model

**Figure 7:**
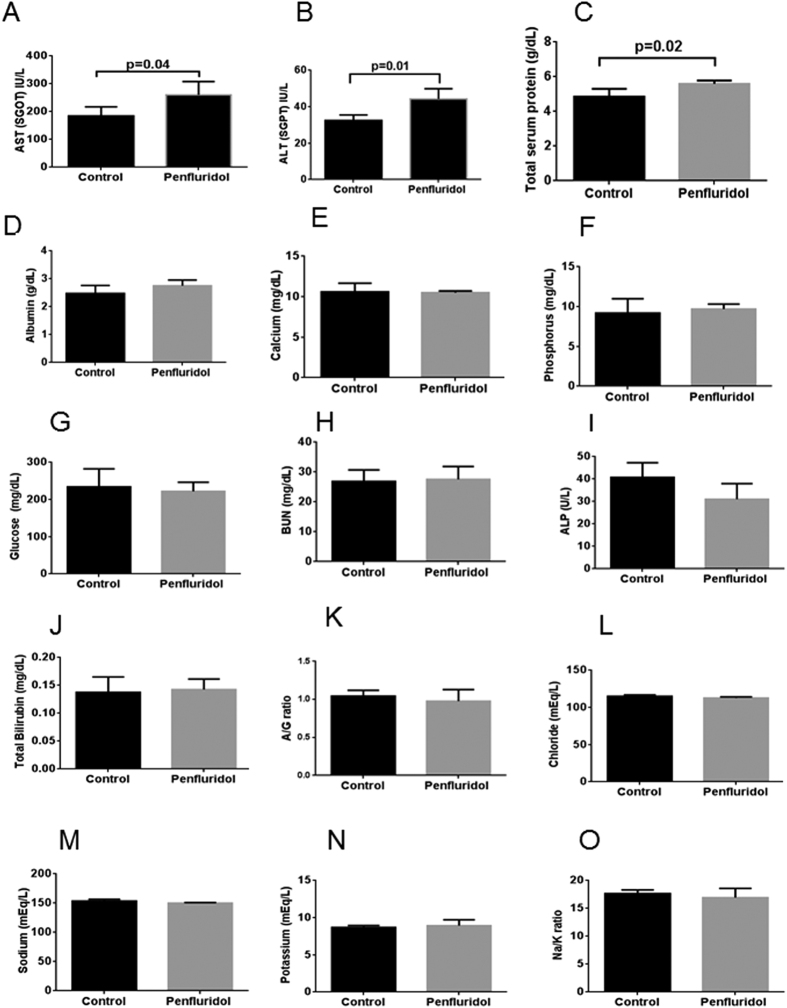
Penfluridol treatment does not cause any major side effect in chronic toxicity model. About 1 × 10^6^ BxPC-3 cells were implanted subcutaneously on right and left flanks of 4–6 week old female athymic nude mice. Once tumor size was around 70 mm^3^, 10 mg/kg penfluridol by oral route was administered every day to mice. After 59 days, mice were sacrificed; plasma was collected and sent to Texas Veterinary Medical Diagnostic Laboratory System, Amarillo, TX for analysis. (**A**) AST (**B**) ALT (**C**) Total serum (**D**) Albumin (**E**) Calcium (**F**) Phosphorus (**G**) Glucose (**H**) BUN (**I**) ALP (**J**) Total Bilirubin (**K**) A/G ratio (**L**) Chloride (**M**) Sodium (**N**) Potassium (**O**) Na/k ratio. Values were plotted as means ± SD.

After establishing the efficacy of penfluridol in inhibiting the growth of pancreatic tumors *in vitro* and *in vivo* by autophagy-mediated apoptosis, we wanted to extensively evaluate the major side effects related with penfluridol treatment in a chronic toxicity model. Hence, in a separate experiment, mice with subcutaneous pancreatic tumors on both the flanks were treated with 10 mg/kg penfluridol by oral gavage for 59 days. After 59 days, comprehensive metabolic and liver function enzymes were evaluated in the blood of control and penfluridol treated mice. Our results showed no significant difference in albumin, calcium, phosphorus, glucose and BUN level in penfluridol treated mice as compared to control mice (Fig. 7D–H). Similarly, we did not observe any significant change in ALP, total bilirubin and A/G ratio as well (Fig. 7I–K). Ionic balance is vital to maintain several important functions in the body, which if disturbed can lead to life threatening diseases. Chronic treatment with penfluridol did not affect important ions like chloride, sodium, potassium and Na/K ratio (Fig. 7L–O). However, we observed modest but clinically irrelevant increase in AST (SGOT), ALT (SGPT) and total serum protein (Fig. 7A–C). Our results suggest that chronic treatment with 10 mg/kg penfluridol by oral route was perhaps not associated with any major toxic side effects.

## Discussion

Our current study established that penfluridol, a drug for schizophrenia, suppressed the growth of pancreatic cancer *in vitro* and *in vivo* by inducing autophagy-mediated apoptosis. Autophagy-mediated apoptosis in different cancer models by anti-cancer agents like cordycepin has been observed recently^12^. A study was published this year showing that thioridazine, an antipsychotic drug induce autophagy-mediated cell death in glioblastoma cells^13^. However, thioridazine was associated with substantial toxicity in clinical setting^9^.

While our work was in progress, Chien *et al.* published a study showing that penfluridol suppress the proliferation of pancreatic cancer cells with IC_50_ ranging between 8.9–36.9 μM^11^. However, in our study, the IC_50_ of penfluridol ranged between 2–7 μM in Panc-1, AsPC-1 and BxPC-3, pancreatic cancer cells. Furthermore, Chien *et al.* demonstrated induction of PP2A (protein phosphatase 2A), a tumor suppressor protein in pancreatic cancer cells by penfluridol treatment, as the main mechanism of the anticancer effects of penfluridol.

Recently, autophagy has emerged as an important target in cancer. It has been argued that inducing autophagy could be a therapeutic strategy for pancreatic cancer^14^. Pancreatic cancer cells have high level of basal autophagy^1^. Our current results indicated that penfluridol treatment induced significant autophagy in AsPC-1, BxPC-3 and Panc-1 cells in a concentration-dependent manner, as evaluated by acridine orange assay as well as by microscopy. Autophagy induction with penfluridol treatment in pancreatic cancer cells was further demonstrated by immunofluorescence and lysotracker assay. One of the hallmarks of autophagy is the accumulation of LC3B and its localization in vesicular structures. We observed that penfluridol treatment enhanced the expression of LC3B and hence induced autophagy in pancreatic cancer cells. Our studies are in agreement with other published studies, which show that agents such as γ-tocotrienol and oridonin exhibit anti-cancer effects by induction of autophagy through upregulation of LC3B^15^. Punctate LC3B formation is an important feature associated with autophagy phenomena^16^. Our studies demonstrated punctate LC3B formation by penfluridol treatment. p62 is another marker of autophagy that selectively gets incorporated into autophagosomes by directly linking to LC3B. We observed an increase in the expression of p62 with penfluridol treatment in pancreatic cancer cells. Although, relationship between expression of p62 and autophagy is controversial, our results are in accordance with other published studies demonstrating increase in p62 expression during autophagy^17^,^18^,^19^,^20^.

Interestingly, role of autophagy in cancer cells is controversial. Several published studies demonstrated that autophagy supports survival of cancer cells^21^. FDA-approved anti-cancer agents like gemcitabine have been shown to induce autophagy to block progression of cancer. However, tumor cells eventually get resistant to gemcitabine^4^. In the current study, we demonstrated that penfluridol treatment resulted in growth suppression of pancreatic cancer *in vitro* and *in vivo* by autophagy-mediated apoptosis. Chloroquine, bafilomycin A1 and 3-methyladenine, known autophagy inhibitors and lysosomotrophic agents, cause an increase in LC3B expression due to blocking of LC3B degradation^22^. In agreement, we observed that penfluridol treatment in combination with chloroquine, bafilomycin or 3-methyladenine exhibited enhanced expression of LC3B when compared to cells treated with penfluridol or inhibitors alone, indicating increased autophagic flux. Bafilomycin and chloroquine treatment block the fusion between autophagosomes and lysosomes, thus inhibit the ultimate step in autophagy. Blocking autophagy using autophagy inhibitors induced less apoptosis by penfluridol treatment in our studies. Similarly, we also observed that inhibiting autophagy using LC3BsiRNA reduced apoptosis by penfluridol treatment. Our study is in agreement with published studies demonstrating that autophagy induction lead to suppression of cancer growth^23^.

We recently reported that 10 mg/kg penfluridol everyday by oral route suppressed breast cancer metastasis to brain, without any signs of toxicity or behavioral side effects^24^. A population based cohort study suggested that use of neuroleptic agents provides protection from various types of cancers like prostate, colon and rectal cancer^25^. In the current study, we observed that 10 mg/kg penfluridol significantly suppressed the growth of xenografts as well as orthotopically implanted pancreatic tumors. Chloroquine, an anti-malarial drug is an established autophagy inhibitor^26^. Penfluridol when combined with chloroquine resulted in decreased efficacy of penfluridol *in vivo* in suppressing the growth of BxPC-3 and AsPC-1 tumor xenografts. These results suggest that the tumor growth suppressive effect of penfluridol was through induction of autophagy-mediated apoptosis. Consistent with our *in vitro* observations, autophagy-mediated pancreatic tumor growth suppression with penfluridol treatment was through apoptosis as demonstrated by western blot, immunohistochemistry and TUNEL staining. On the other hand, a study suggested that the anti-cancer effect of penfluridol was through dysregulation of cholesterol homeostatsis^27^. Safety profile of 10 mg/kg penfluridol in a chronic study performed by us indicated that penfluridol is perhaps safe to use. Further detailed toxicological evaluation of penfluridol is thus warranted.

Overall, our studies established autophagy-mediated anti-tumor effects of penfluridol in pancreatic cancer. To the best of our knowledge, this is the first report of anti-tumor activity of penfluridol through the induction of autophagy-mediated apoptosis. Taken together, our results lay the foundation for repurposing penfluridol as a drug for treatment of pancreatic cancer, which otherwise is highly resistant to chemotherapy and difficult to treat.

## Materials and Methods

### Ethics Statement

All the animal experiments have been conducted in accordance with the ethical standards and according to approved protocol by Institutional Animal Care and Use Committee (IACUC), Texas Tech University Health Sciences Center, Texas.

### Cell culture

BxPC-3 and AsPC-1 human pancreatic cancer cell lines were procured from ATCC, Manassas, VA. Panc-1 cells were kind gift from Dr. Thomas L. Brown (Wright State University, Dayton, OH). All the cell lines were authenticated by institutional core laboratory using STR method. Cells were maintained and cultured as we have described previously^28^. All the cells used in this study were within twenty passages after receipt or resuscitation.

### Cytotoxicity Studies

Pancreatic cancer cells were plated at a density of 3000–5000 cells per well in 96 well plates. After overnight incubation, penfluridol (Sigma-Aldrich, St. Louis, MO) was added in different concentrations. After desired durations of treatment (24, 48 and 72 h), cells were fixed by using ice cold 10% trichloroacetic acid. Plates were washed gently with water and stained with sulphorhodamine B (SRB) dye. After staining, plates were washed with 1% acetic acid solution followed by measuring optical density in 10 mM Tris-base solution using plate reader (BioTek Instruments, VT) and as described by us previously^24^.

### Annexin V/FITC apoptosis assay

Apoptosis assay was performed using a commercially available kit and as per manufacturer’s instruction (BD Biosciences, San Jose, CA, USA). Approximately 0.3 × 10^6 ^cells were plated in each well of 6 well plate and left overnight in the incubator. Penfluridol was added in required concentration for 24 h. After 24 h of treatment, cells were harvested by trypsinization and suspended in PBS. Cells were washed and suspended in 150 μl of binding buffer. 4 μl Annexin-V FITC and 4 μl propidium iodide was added and incubated for 20 minutes in dark. Volume was adjusted to 300 μL by adding binding buffer. Samples were analyzed by flow cytometer after vortexing (Accuri C6, Ann Arbor, MI, USA) and as described by us previously^29^.

### Western blotting

Panc-1, BxPC-3 and AsPC-1 cells were treated with varying concentration of penfluridol for 24 h. Cells were collected and washed with phosphate-buffered solution twice. Lysis was performed as described by us previously^28^. Protein content was determined using Bradford reagent. 20–60 μg of protein was subjected to SDS gel electrophoresis and resolved proteins were transferred to PVDF membrane followed by immunoblotting and as described by us previously^28^. The membranes were probed for primary antibodies against LC3B, p62, cleaved caspase 3, cleaved PARP as well as actin. Antibodies against LC3B (Cat.#3868S), p62 (Cat.#5114S), cleaved caspase 3 (Cat.#9661S) and cleaved PARP (Cat.#9541S) were purchased from Cell Signaling Technology, Danvers, MA. Actin (Cat.#A5441) antibody was purchased from Sigma Aldrich, St. Louis, MO. The membranes were developed as described by us previously^30^,^31^,^32^,^33^,^34^.

### Acridine orange assay

Panc-1, AsPC-1 and BxPC-3 cells were plated at a density of 0.3 × 10^6 ^cells per well in six well plates and was allowed to attach overnight. Cells were treated with or without penfluridol for 24 h. Cells were trypsinised, washed with PBS and suspended in binding buffer. Cells were incubated with 0.4 μg/ml acridine orange (Sigma-Aldrich, St. Louis, MO) for 15 minutes. Readings were taken using C6 flow Accuri flowcytometer (Ann Arbor, MI) and as described by us previously^18^.

### Immunofluorescence analysis

AsPC-1 cells were plated in a 24-well plate on a coverslip at a density of 0.1 × 10^6^ cells/well and allowed to attach overnight. Attached cells were treated with 2.5 μM penfluridol for additional 24 h. The cells were fixed with formalin and permeabilized using Triton-X100 solution. 1% BSA with goat serum was used for blocking after which cells were incubated overnight with primary antibody for LC3B (1:200). Next day, cells were washed and incubated with AlexaFluor 488 secondary antibody (Invitrogen, Carlsbad, CA). After washing, the coverslips were mounted on slides (mounting media with DAPI) and images were taken using fluorescence microscope (Olympus, Center Valley, PA) and as described by us before 24.

### Acridine orange staining for microscopy

AsPC-1 cells at a density of 0.3 × 10^6^ cells per well was plated in a six well plates. Cells were allowed to attach overnight after which cells were treated with 2.5 and 5.0 μM penfluridol for 24 h. Media was replaced with 1ml of PBS in each well. 2–3 drops of NucBlue live cell stain (Life technologies, Eugene, OR) was added to each well for 20 minutes. Acridine orange at concentration of 0.4 μg/ml was added and images were taken immediately using florescence microscope (Olympus, Center Valley, PA).

### Inhibitors treatment

AsPC-1 cells were plated at a density of 0.3 × 10^6 ^cells per well in a six well plate. After overnight incubation, cells were pretreated for 3 h with 10 nM bafilomycin A1, 5 mM 3-methyladenine and 5 μM as well as 10 μM chloroquine. Bafilomycin A1 and 3- methyladenine were purchased from Sigma-Aldrich, St. Louis, MO. Chloroquine diphosphate salt was purchased from MP Biomedicals, LLC, Solon, OH. After pretreatment with inhibitors, cells were treated with 5 μM penfluridol for 24 h and processed for western blotting as explained above.

### LC3B silencing

AsPC-1 cells were transfected with LC3B siRNA (Cell Signaling Technologies, Danvers, MA) using siPORT (Ambion Inc, Austin, TX) transfection reagent as per manufacturer’s protocol (Cell Signaling Technologies, Danvers, MA). Cells were transfected with 100 nM LC3B siRNA or scrambled siRNA and after 24 h post transfection, cells were treated for additional 24 h with 5 μM penfluridol. The cells were collected after treatment and processed for western blot analysis as described by us before^18^.

### Lysotracker assay

AsPC-1 cells at a density of 0.3 × 10^6^ per well was plated in a six well plate and allowed to attach overnight. Cells were pretreated with or without 10 μM chloroquine for 3 h. After pretreatment, cells were treated with 5 μM of penfluridol for 24 h. Media was removed, cells were washed and 1 ml of PBS was added to each well of the plate. 2–3 drops of NucBlue live cell stain (Life technologies, Eugene, OR) was added to each wells for 20 minutes. 50 nM lysotracker green DND-26 (cell signaling, Danvers, MA) was directly added to the plate and images were taken immediately using florescence microscope (Olympus, Center Valley, PA).

### Subcutaneous tumor implantation

Athymic nude mice (4–6 week old) were purchased from Harlan Laboratory (Livermore, CA). The use and treatment of athymic nude mice is approved from Institutional Animal Care and Use Committee (IACUC), Texas Tech University Health Sciences Center, Amarillo, Texas. All the experiments were performed under the strict compliance and regulations. 1 × 10^6^ AsPC-1 and BxPC-3 cells in 1:1 mixture of PBS and matrigel were implanted in right and left flank of mice respectively. Once tumor volume was around 70 mm^3^, mice were randomly divided into four groups with 5 mice in each group. Group I served as control and received the vehicle only. Group II received 10 mg/kg penfluridol by oral gavage everyday whereas group III received, 50 mg/kg chloroquine (i.p) every day. Group IV received 10 mg/kg penfluridol and 50 mg/kg chloroquine respectively every day. Penfluridol stock was prepared in DMSO, which was further diluted in water/PEG300/ethanol/2% acetic acid in 8:3:3:1 v/v^35^. Chloroquine stock was prepared in water. Tumor volume was measured twice a week till day 27 by using vernier caliper as described by us before^36^. At day 27, mice were humanely sacrificed and tumors were removed aseptically. A part of tumor was snap frozen for western blotting and other part was fixed in formalin for immunohistochemistry.

### Immunostaining of tissue sections

The immunohistochemistry (IHC) was performed as previously described by us^28^,^29^. Briefly, fixed tumor tissues were dehydrated and embedded in paraffin. Tissues were sectioned into 5 μm thick sections using microtome (Leica Microsystems Inc., Buffalo Grove, IL). The sections were deparaffinized as well as rehydrated using xylene, ethanol and double-distilled water washes. Unmasking of antigen was done by boiling the sections in 10 mM sodium citrate buffer (pH 6.0). Sections were washed and incubated in 3% hydrogen peroxide solution. The sections were blocked with 5% goat serum and incubated with primary antibodies for cleaved caspase 3 (1:300), LC3B (1:1000) and p62 (1:100) overnight at 4 °C. Next day the slides were stained using Ultravision ONE HRP polymer kit as per the manufacturer’s instructions (Thermofisher scientific, Fremont, CA). Counterstaining of sections was performed with Mayer’s hematoxylin and dehydrated. The slides were mounted using Permount (Fisher scientific, Fair Lawn, NJ) and imaged using Olympus microscope (Olympus America Inc, Center Valley, PA). Antibodies for cleaved caspase 3 (Cat.#9661S), LC3B (Cat.#3868S) and p62 (Cat.#5114S) were purchased from Cell Signaling. TUNEL assay was carried out according to manufacturer’s protocol (TUNEL assay kit was purchased from Calbiochem, San Diego, CA, USA).

### Orthotopic tumor model

Female athymic nude mice (4–6 weeks old) from Harlan Laboratories (Livermore, CA) were used for orthotopic injection. The experiments were conducted in strict compliance with the regulations of Institutional Animal Care and Use Committee (IACUC), Texas Tech University Health Sciences Center. Mice were anesthetized by using isoflorane and a small incision was made to implant stably luciferase-expressing Panc-1 (PanC-1-luc) cells orthotopically in the pancreas. 20 μl of PBS suspension containing 1 × 10^6^ exponentially growing Panc-1- luc cells were injected into the subcapsular region on the pancreas using a 30-gauge sterile needle. The peritoneum and skin incisions were closed sequentially with absorbable suture. Pain killer was given to the animals at every 8 h for initial 2 days. Mice were closely monitored twice a day until they recovered completely from the surgery stress. Mice were imaged on the day of injection using IVIS (Caliper Life Sciences) to have basal luminescence value after injecting luciferin (3 mg/mouse, ip). On day 9^th^ after the surgical implantation of Panc-1-luc cells, mice were divided randomly into 2 groups with 6 mice in each group. The treatment group received 10 mg/kg penfluridol by oral gavage whereas control group received vehicle only. Tumor luminescence was measured thrice a week. At the end of the experiment, mice were sacrificed; pancreases with tumors were excised, snap frozen as well as processed for immunohistochemistry.

### Safety profile study

Athymic nude mice (4–6 week old) were obtained from Harlan laboratory (Livermore, CA). The animal experiments were conducted in strict compliance with the regulations and approved protocols by the Institutional Animal Care and Use Committee (IACUC), Texas Tech University Health Sciences Center. 1 × 10^6^ BxPC-3 cells in 1:1 mixture of PBS and matrigel were implanted in right and left flank of mice. Once tumor volume was around 70 mm^3^, mice were randomly divided into two groups with 10 mice in each group. Mice were administered 10 mg/kg penfluridol by oral gavage every day for 59 days. Control mice received the vehicle only. At the end of 59 days, the mice were sacrificed and blood was extracted and harvested to separate plasma. Chemistry panel with 4 plasma samples from each group was performed at Texas Veterinary Medical Diagnostic Laboratory System, Amarillo, TX.

### Statistical Analysis

Prism 6.0 software was used for all the statistical analysis (GraphPad software Inc., San Diego, CA). Results are represented as means ± standard deviation (SD) or standard error of means (SEM). Statistical significance was analyzed using Student’s *t*-test.

## Additional Information

**How to cite this article**: Ranjan, A. and Srivastava, S. K. Penfluridol suppresses pancreatic tumor growth by autophagy-mediated apoptosis. *Sci. Rep.* **6**, 26165; doi: 10.1038/srep26165 (2016).
